# The effect of childhood trauma and trauma-focused psychotherapy on blood expression of MED22 in patients with major depressive disorder

**DOI:** 10.1192/j.eurpsy.2022.226

**Published:** 2022-09-01

**Authors:** A. Minelli, E. Maffioletti, R. Carvalho Silva, A. Cattaneo, G. Perusi, M. Bortolomasi, M. Gennarelli

**Affiliations:** 1 University of Brescia, Department Of Molecular And Translational Medicine, Brescia, Italy; 2 University of Brescia, Department Of Molecular And Translational Medicine, Verona, Italy; 3 IRCCS Fatebenefratelli San Giovanni di Dio, Biological Psychiatry Unit, Brescia, Italy; 4 Psychiatric Hospital, Villa Santa Chiara, Verona, Italy

**Keywords:** major depressive disorder, Childhood Trauma, Psychotherapy, Blood Expression

## Abstract

**Introduction:**

The only available genome-wide study (Minelli et al., 2018) indicated an association between the neglect CT and MED22, a transcriptional factor gene.

**Objectives:**

To verify how the dysregulation of MED22 could be affected by environmental and genetic factors, we carried out an analysis on these components and a longitudinal study concerning the effect of trauma-focused psychotherapy in MDD patients that experienced CT.

**Methods:**

On a large mRNA sequencing dataset including 368 MDD patients we computed the genetic (GReX) and the environmental (EReX) components affecting gene expression in relation to CT. Furthermore, we measured the expression of MED22 in 22 MDD patients treated with trauma-focused psychotherapy.

**Results:**

The dissection of *MED22* expression profiles revealed an association of neglect with environmental and genetic components (p=6x10^-3^ p=2.6x10^-4^). Furthermore, in an independent cohort of 177 controls, we also observed a significant association between cis-eSNPs of *MED22* and higher neuroticism scores (best p-value: 0.00848) that are usually associated with a decreased amount of resilience to stress events. Finally, the results of psychotherapy revealed a reduction of depressive symptomatology (p<0.001) and 73% of patients resulted responders at the follow-up visit. MED22 expression during psychotherapy showed a change trend (p=0.057) with an interaction effect with response (p=0.035). Responder and non-responder patients showed MED22 expression differences at different trauma-focused psychotherapy timepoints (p=0.15; p=0.012) and at the follow-up (p=0.021).

**Conclusions:**

Our results provide insights suggesting that some biological and clinical consequences of CT depend on genetic background and environmental factors that could induce vulnerability or resilience to stressful life events.

**Disclosure:**

No significant relationships.

